# Tackling *Acinetobacter baumannii*

**DOI:** 10.3390/jcm12165168

**Published:** 2023-08-08

**Authors:** Guido Granata, Fabrizio Taglietti, Nicola Petrosillo

**Affiliations:** 1 Clinical and Research Department for Infectious Diseases, National Institute for Infectious Diseases L. Spallanzani, IRCCS, 00149 Rome, Italy; guido.granata@inmi.it (G.G.);; 2 Infection Prevention & Control/Infectious Disease Service, Fondazione Policlinico Universitario Campus Bio-Medico, 00127 Rome, Italy

Globally, multidrug-resistant (MDR) bacteria represent a menace to public health. Recently, the World Health Organization recognized the ubiquitous, non-fermenting, rod-shaped Gram-negative coccobacillus *Acinetobacter baumannii* as a critical MDR bacteria [1].

*Acinetobacter baumannii* can be considered an opportunistic pathogen, predominantly affecting critically ill patients or patients with immune system deficiency [1]; as a consequence, *Acinetobacter baumannii* frequently causes healthcare-associated infections, including pneumonia, bloodstream infection, meningitis, urinary tract and wound infection.

In this Editorial, we present a bundle of recent articles on different aspects of the *Acinetobacter baumannii* threat, including the current nosocomial burden, prevention of transmission in hospitals, and clinical management.

Recently, Jean et al. provided a picture of the current burden of hospital-acquired pneumonia due to MDR bacteria worldwide [2]. The authors reviewed the available literature to identify the organisms most commonly implicated in hospital-acquired pneumonia, as well as the risk factors and the most effective antibiotic options.

They found that the most frequently isolated bacteria causing hospital-acquired pneumonia was *Staphylococcus aureus*, followed by *Pseudomonas* spp. and *Acinetobacter* spp. [2].

In several Asian countries, including China, Thailand and Taiwan, Gram-negative bacteria are currently the most common cause of hospital-acquired pneumonia, with a trend towards an increase in MDR *Acinetobacter baumannii* [2].

Among the risk factors associated with the onset of MDR *Acinetobacter baumannii* pneumonia, the authors identified a higher co-morbidity index, prolonged hospital stay, previous administration of broad-spectrum antibiotics and intensive care unit stay [2].

The burden of respiratory tract infections due to *Acinetobacter baumannii* in the intensive care units was also confirmed by a prospective respiratory infections’ surveillance study at a 30-bed intensive care unit in Poland [3]. Duszynska et al. assessed the frequency, etiology, mortality, and additional costs of respiratory tract infections. Overall, pneumonia was found in 136/578 patients (24%). The authors reported non-ventilator hospital-acquired pneumonia and ventilator-associated pneumonia rates of 9.0 and 8.65/100 admissions, respectively [3]. Alarmingly, MDR *Acinetobacter baumannii* was the most frequently isolated bacteria. During the study, the reported mortality rate among patients with non-ventilator hospital-acquired pneumonia and ventilator-associated pneumonia was 44% and 38%, respectively [3].

*Acinetobacter baumannii* spread in the intensive care setting represents a main issue that the COVID-19 pandemic has worsened. In fact, a recent observational, multicenter, retrospective study performed in Italy reported an increased burden of MDR *Acinetobacter baumannii* in intensive care units during the COVID-19 pandemic [4].

Overall, the study included 913 COVID-19 patients admitted to the intensive care units; of them, 176 (19%) became positive for carbapenem-resistant *Acinetobacter baumannii* during the intensive care unit stay, either colonization (47/176 patients, 26.7%) or infection (129/176 patients, 73.2%). As a further issue, the observed mortality rate in carbapenem-resistant *Acinetobacter baumannii* patients was extremely high, i.e., 64.7% [4].

In addition to ventilator-associated pneumonia or bloodstream infections, hospital associated urinary tract infections can be caused by *Acinetobacter baumannii*.

A prospective study performed over six years in a large private hospital in Greece investigated the correlation between the incidence rate of catheter-associated urinary tract infections, specific infection-control measures and the incidence rate of MDR bacteremia [5]. Infection control measures included the active surveillance of MDR pathogens, implementation of an intervention bundle for urinary catheters, promotion of hand hygiene before and after providing healthcare to patients, and pharyngeal, axillary-rectal and nasal screening, followed by the isolation of MDR carrier patients.

This study provided comforting results. The intervention bundle was effective, determining a decrease in the incidence of catheter-associated urinary tract infections [5]. The decrease in catheter-associated urinary tract infections rate also determined a decrease in the incidence of urinary tract infections due to MDR *Acinetobacter baumannii* [5].

In almost all the articles dealing with *Acinetobacter baumannii* the most concerning issue is represented by the resistance to most antibiotics, including carbapenems and, in some cases, polymixins and, as a consequence, the limited therapeutical options [1].

At present, possible antibiotic options with in vitro activity against MDR *Acinetobacter baumannii* are restricted to few antimicrobial classes, i.e., aminoglycosides, polymyxins, tetracyclines and phosphonic antibiotics. Unfortunately, these antimicrobials have important limitations, including toxicities, site-specific pharmacokinetics and emergence of resistance. A meta-analysis by Huang et al. compared the efficacy and the safety of colistin monotherapy versus colistin and meropenem combination for the treatment of patients with MDR *Acinetobacter baumannii* infection [6]. The meta-analysis included three randomized controlled trials and seven retrospective studies. The authors found that monotherapy shows clinical improvements, and reduced hospital mortality and nephrotoxicity comparable to the combination therapy with colistin and meropenem [6].

Recently, two novel antimicrobials, cefiderocol and sulbactam-durlobactam, have been considered for the treatment of infections due to MDR *Acinetobacter baumannii* [7].

Cefiderocol showed valuable activity against *Acinetobacter baumannii*, but unfortunately the CREDIBLE trial reported variable in vivo efficacy [8].

Sulbactam-durlobactam showed in vitro potency against *Acinetobacter baumannii* isolates, and possesses valuable pharmacokinetic and pharmacodynamic profiles for the treatment of bloodstream infections and pneumonia [7]. Therefore, great hope is being placed in this compound as a new option to treat patients with infections due to MDR *Acinetobacter baumannii*.

Recently, a multicentre, randomised, phase 3 clinical trial was performed to evaluate the efficacy and safety of sulbactam-durlobactam in patients with bloodstream infections or hospital-associated bacterial pneumonia due to *Acinetobacter baumannii* [9].

The trial aimed to assess the non-inferiority of sulbactam–durlobactam versus colistin, both given with imipenem–cilastatin for the treatment of infections caused by *Acinetobacter baumannii* [9]. The trial randomized 181 patients, and 125 patients were assessed for primary efficacy analysis. Sulbactam–durlobactam achieved statistical non-inferiority to colistin in the 28-day all-cause mortality efficacy endpoint. Sulbactam–durlobactam mortality was 19.0% compared to 32.3% in the colistin arm (treatment difference of −13.2%; 95% confidence interval: −30.0, 3.5) [9].

However, although these findings confirmed sulbactam-durlobactam as a promising treatment, important limitations should be highlighted.

Most of the patients enrolled had *Acinetobacter baumannii* isolated in sputum or in the respiratory tract. As polymicrobial infections were reported in almost 30% of the patients included, the isolation of *Acinetobacter baumannii* in respiratory samples could represent a colonization. Therefore, it cannot be excluded that a relevant percentage of the included cases may represent *Acinetobacter-baumannii*-colonized patients, with an ongoing infection due to other bacteria. Further randomized clinical trials are needed to confirm the validity of sulbactam-durlobactam to treat *Acinetobacter baumannii* infections [7].

The differences between monomicrobial and polymicrobial *Acinetobacter baumannii* bacteremia was recently studied by Wang et al. in a retrospective observational study [10]. Overall, the study included 379 patients with *Acinetobacter baumannii* bacteremia. Among them, 290 patients (76.5%) had monomicrobial bacteremia and 89 patients (23.5%) had polymicrobial bacteremia, including *Acinetobacter baumannii*. No significant difference in mortality at two weeks was reported between patients with monomicrobial and polymicrobial bacteremia (26.9% versus 29.2%, respectively); however, higher mortality rates were found among polymicrobial bacteremic patients with concomitant isolation of *Escherichia coli*, *Pseudomonas aeruginosa*, and *Enterobacter* spp. The logistic regression analysis demonstrated that appropriate antibiotic treatment was independently associated with a decrease in mortality [10].

To conclude, the global spread of MDR *Acinetobacter baumannii* strains causes severe infections, with restricted antibiotic options and high mortality rates.

More than ever, infection-control measures are needed to minimize the spread of *Acinetobacter baumannii* in the hospitals. Surveillance, infection prevention and control, as well as knowledge of the mechanisms of resistance and novel antimicrobial compounds against *Acinetobacter baumannii,* are the main means of tackling this worldwide menace.

## References

[1] European Centre for Disease Prevention and Control Carbapenem-Resistant Acinetobacter baumannii in Healthcare Settings. https://www.ecdc.europa.eu/sites/default/files/media/en/publications/Publications/8-Dec-2016-RRA-Acinetobacter%20baumannii-Europe.pdf.

[2] Jean S.S., Chang Y.C., Lin W.C., Lee W.S., Hsueh P.R., Hsu C.W. (2020). Epidemiology, Treatment, and Prevention of Nosocomial Bacterial Pneumonia. J. Clin. Med..

[3] Duszynska W., Idziak M., Smardz K., Burkot A., Grotowska M., Rojek S. (2022). Frequency, Etiology, Mortality, Cost, and Prevention of Respiratory Tract Infections—Prospective, One Center Study. J. Clin. Med..

[4] Montrucchio G., Corcione S., Lupia T., Shbaklo N., Olivieri C., Poggioli M., Pagni A., Colombo D., Roasio A., Bosso S. (2022). The Burden of Carbapenem-Resistant *Acinetobacter baumannii* in ICU COVID-19 Patients: A Regional Experience. J. Clin. Med..

[5] Papanikolopoulou A., Maltezou H.C., Stoupis A., Kalimeri D., Pavli A., Boufidou F., Karalexi M., Pantazis N., Pantos C., Tountas Y. (2022). Catheter-Associated Urinary Tract Infections, Bacteremia, and Infection Control Interventions in a Hospital: A Six-Year Time-Series Study. J. Clin. Med..

[6] Huang C., Chen I., Tang T. (2022). Colistin Monotherapy versus Colistin plus Meropenem Combination Therapy for the Treatment of Multidrug-Resistant *Acinetobacter baumannii* Infection: A Meta-Analysis. J. Clin. Med..

[7] Granata G., Taglietti F., Schiavone F., Petrosillo N. (2022). Durlobactam in the Treatment of Multidrug-Resistant *Acinetobacter baumannii* Infections: A Systematic Review. J. Clin. Med..

[8] Bassetti M., Echols R., Matsunaga Y., Ariyasu M., Doi Y., Ferrer R., Lodise T.P., Naas T., Niki Y., Paterson D.L. (2021). Efficacy and safety of cefiderocol or best available therapy for the treatment of serious infections caused by carbapenem-resistant Gram-negative bacteria (CREDIBLE-CR): A randomised, open-label, multicentre, pathogen-focused, descriptive, phase 3 trial. Lancet Infect. Dis..

[9] Kaye K.S., Shorr A.F., Wunderink R.G., Du B., Poirier G.E., Rana K., Miller A., Lewis D., O’Donnell J., Chen L. (2023). Efficacy and safety of sulbactam-durlobactam versus colistin for the treatment of patients with serious infections caused by *Acinetobacter baumannii-calcoaceticus* complex: A multicentre, randomised, active-controlled, phase 3, non-inferiority clinical trial (ATTACK). Lancet Infect. Dis..

[10] Wang Y.C., Ku W.W., Yang Y.S., Kao C.C., Kang F.Y., Kuo S.C., Chiu C.H., Chen T.L., Wang F.D., Lee A.Y. (2020). Is Polymicrobial Bacteremia an Independent Risk Factor for Mortality in *Acinetobacter baumannii* Bacteremia?. J. Clin. Med..

